# Incidence, Time Trends and Geographical Distribution of Leukemia and Multiple Myeloma in Golestan Province, Northern Iran, 2004–2017

**DOI:** 10.34172/aim.2022.59

**Published:** 2022-06-01

**Authors:** Nastaran Jafari-Delouei, Mohammad Naimi-Tabiei, Mehran Farajollahi, Seyed Mehdi Sedaghat, Seyyedreza Khandoozi, Fatemeh Ghasemi-Kebria, Roshan Dinparastisaleh, Amirhoushang Pourkhani, Gholamreza Roshandel

**Affiliations:** ^1^Golestan Research Center of Gastroenterology and Hepatology, Golestan University of Medical Sciences, Gorgan, Iran; ^2^Cancer Research Center, Golestan University of Medical Sciences, Gorgan, Iran; ^3^Deputy of Public Health, Golestan University of Medical Sciences, Gorgan, Iran; ^4^Outcomes After Critical Illness and Surgery Group, Johns Hopkins University, Division of Pulmonary and Critical Care Medicine, Johns Hopkins School of Medicine, Baltimore, MD, USA

**Keywords:** Epidemiology, Golestan, Iran, Leukemia, Multiple myeloma

## Abstract

**Background:**

Leukemia and multiple myeloma (MM) are the most common hematologic malignancies in Iran. This paper describes the geographic and temporal changes in their incidence in Golestan, northern Iran.

**Methods:**

Data on cases of leukemia and MM during 2004–2017 were obtained from the Golestan Population-based Cancer Registry (GPCR). The GPCR is a dynamic database of Golestan residents diagnosed with primary cancers. Age-standardized incidence rates (ASRs) (per 100000) of leukemia and MM were calculated using direct standardization method considering the world standard population. We used Joinpoint regression to assess incidence trends using the average annual percent change (AAPC).

**Results:**

In total, 2119 new cases of leukemia and MM were registered by the GPCR during 2004–2017. The ASRs of leukemia were 9.71 and 6.70 in males and females, respectively, while the rates were lower for MM: 2.66 and 1.97 in males and females, respectively. The incidence rates of leukemia suggested an increasing trend in urban population (AAPC=2.73; *P* value=0.154), while in rural area, the incidence rates were slightly decreasing (AAPC=- 0.73; *P* value=0.658). There were high incidence areas of leukemia in the central and western regions of Golestan.

**Conclusion:**

Our results suggested high incidence rates of leukemia and MM in the Golestan province. We also found geographical diversities and increasing trends in the incidence of leukemia in the urban population. Exposure to occupational and environmental carcinogens including pesticides may partly explain high rates and the observed trends. Further investigations should be considered to clarify these points in our population.

## Introduction

 Cancer, previously the leading cause of death in most of the developed countries will be a major cause of morbidity and death in the near future in different part of the world, irrespective of the status of resources.^1^ Cancer is the leading cause of mortality in high-income countries and the second cause of mortality in the developing world.^2^ Hematologic malignancies comprise a collection of heterogeneous diseases characterized by the uncontrolled growth of hematopoietic cells. Different factors have been proposed to play a role in the development of hematologic malignancies including infectious diseases (e.g., HTLV-1 and adult T-cell leukemia/lymphoma), autoimmune disorders (e.g., rheumatoid arthritis, systemic lupus erythematous and Sjogren syndrome) or positive family history.^3^

 Hematologic malignancies include different disorders some of which are progressive, malignancies of the blood-forming organs, characterized by distorted proliferation and development of leukocytes and their precursors in the blood and bone marrow, and others are due to lymphatic tissue involvement.^4^ The three major types of hematologic malignancies are leukemia, lymphoma, and plasma cell disorders.^5^ Leukemia is a malignant proliferation of hematopoietic cells in the bone marrow followed by dissemination into the blood and infiltration into soft tissue.^6^ Extramedullary manifestations may occur in different organs (e.g. skin), following a generalization phase in the bone marrow and the subsequent dissemination of cancer cells in the peripheral blood.^6^ Leukemia may be classified based on the its biological behavior and the immunophenotypical, morphological, and cytogenetic characteristics of neoplastic cells in chronic and acute, myeloid or lymphocytic forms of disease.^7,8^ Acute leukemia has been classified by the French‐American‐British (FAB) Cooperative Group. The FAB classification of acute lymphocytic leukemia includes three subtypes (L_1_–L_3_), while acute myeloid leukemia is divided into 8 subtypes (M_0_–M_7_).^9^ Multiple myeloma (MM) is the second most common hematologic malignancy. It is described in the spectrum of plasma cell dyscrasias, ranging from monoclonal gammopathy of unknown significance to overt plasma cell leukemia and extramedullary myeloma. MM may result in significant morbidity due to its effects on end-organ damage and older population involvement.^10^ After decades of virtually no improvement, the MM survival rate has seen major progress in the last 10 years, even 2- to 3-fold in younger patients.^11^ According to GLOBOCAN 2018 estimates of global cancer incidence, leukemia and MM with 437.033 and 159.985 new cases per year, respectively, are amongst the commonly diagnosed malignancies in the world.^12^ The age-standardized incidence rates (ASRs) of leukemia and MM are higher among males, but in North America, Australia, New Zealand and Europe, the male preponderance may be even more remarkable.^12^ Leukemia ranked 7th among the most commonly diagnosed cancers in Iran with 2,174 new cases per year (4.3% of all cases) and a higher ASR in males (8.6 per 100 000) versus females (5.9 per 100 000).^12^

 Golestan is one of the 31 provinces, located in Northern Iran. As a voting member of the International Association of Cancer Registries (IACR), the Golestan Population-based Cancer Registry (GPCR), has been providing high-quality reports on Golestan cancer statistics, since 2004. Previous GPCR reports revealed higher rates of leukemia in the Golestan province, compared with other provinces.^13^ In this paper, we aimed to present an updated report on incidence rates and epidemiology trends of leukemia and MM in the Golestan province of Iran.

## Materials and Methods

 This study was conducted on primary incidence data of leukemia and MM in Golestan, Northern Iran. Golestan is one of the 31 provinces of Iran with about 1.9 million population in 2016 (2.3% of total population of Iran) and an area of about 20 000 km.^2^

 The GPCR, a population-based cancer registry, covers the total population of the Golestan province. The GPCR is a dynamic database of Golestan and territory residents, who were diagnosed with primary cancers (alive or dead). The GPCR database provides demographic, geographic, and clinical information, including the patient’s gender, diagnosis year, age at diagnosis, and residence place as well as the tumor characteristics. Data of the GPCR were collected from all the private and public healthcare centers throughout the Golestan province,and the deputy of health of the Golestan University of Medical Science (GOUMS) sends regular updates on cancer-related deaths.^14^ Additionally, we collected data from sources outside of Golestan to minimize underestimation of new cases due to potential referral to neighboring provinces (e.g. Khorasan Razavi and Tehran).We used the third version of the International Classification of Diseases for Oncology (ICD-O-3) system for coding tumor characteristics.^15^

 For this study, we examined the data on the incidence of leukemia and MM in the GPCR between 2004 and 2017. We used the CanReg-5 software^16,17^ to calculate the age-specific rates, crude rates and the ASRs.^18^ ASRs were calculated by direct standardization method using the 18 age groups world standard population. All rates were presented per 100 000 person-years. Joinpoint regression analysis was used to assess time trends during the study period^19^ using the average annual percent change (AAPC) and its corresponding 95% confidence intervals (CIs).

## Results

 During the study period (2004-2017), a total of 2119 new cases of leukemia and MM were registered in the GPCR with a mean (SD) age of 46.8 (23.9) years, at diagnosis. Of these, 1697 were diagnosed with leukemia with a mean (SD) age of 43.5 (24.7) years, and 422 were diagnosed with MM with a mean (SD) age of 60.1 (13.7) years.

**Figure 1:**
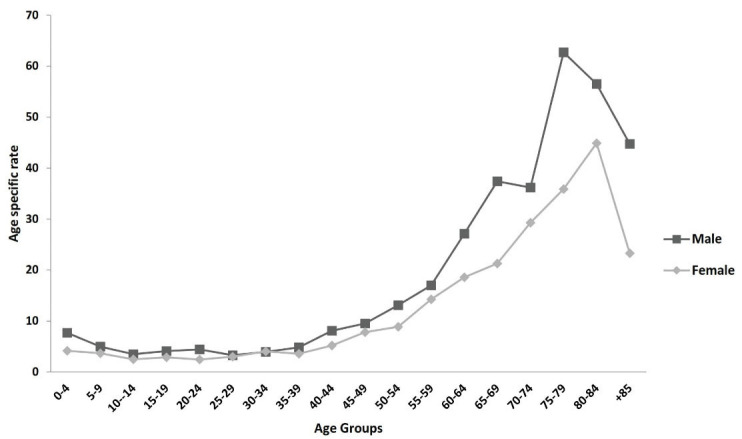
Age Specific Incidence Rate (Per 100 000 Person-Year) of Leukemia in Golestan, Iran, during 2004–2017.

 The number (crude rate; ASR) of leukemia cases were 993 (8.16; 9.71) and 704 (5.77; 6.70) per 100 000 person-year in males and females, respectively. The number (crude rate; ASR) of MM cases were 238 (1.96; 2.66) and 184 (1.51; 1.97) per 100 000 person-year in males and females, respectively (Table 1). Table 2 shows the number, crude rate, ASR and 95% CI of ASR for leukemia in Golestan by incidence year and sex. The number, crude rate, ASR, and 95% CI of ASR for MM and different types of leukemia in the Golestan province by incidence, year, and gender are shown in Table S1 (see Supplementary file 1). Figure 1 shows the age-specific incidence rates of leukemia by gender. As shown in Figure 1, the leukemia incidence rate peaks at the age group 75-79 and 80-84 in males and females, respectively.

**Table 1:** Number, Crude Rate and ASR and 95% Confidence Interval (Per 100 000 Person-Year) of Leukemia and Multiple Myeloma by Morphology and Gender in Golestan, Iran during 2004 to 2017

	**Male**					**Female**					**All**				
	**Number**	**Crude**	**ASR**	**ASR-L**	**ASR-U**	**Number**	**Crude**	**ASR**	**ASR-L**	**ASR-U**	**Number**	**Crude**	**ASR**	**ASR-L**	**ASR-U**
Lymphoid-leukemia	436	3.58	4.34	3.91	4.77	246	2.02	2.43	2.12	2.74	682	2.8	3.38	3.13	3.63
Myeloid-leukemia	274	2.25	2.6	2.29	2.91	224	1.84	2.11	1.82	2.4	498	2.04	2.35	2.13	2.57
Unspecified-leukemia	283	2.33	2.78	2.45	3.11	234	1.92	2.16	1.87	2.45	517	2.12	2.46	2.24	2.68
MM	238	1.96	2.66	2.31	3.01	184	1.51	1.97	1.68	2.26	422	1.73	2.31	2.07	2.55

ASR, Age-standardized incidence rate; ASR-L, Lower limit of 95% CI; ASR-U, Upper limit of 95% CI; MM, multiple myeloma.

**Table 2:** Number, Crude Rate and ASR and 95% Confidence Interval (Per 100 000 Person-Year) of Leukemia by Incidence Year and Gender in Golestan, Iran, 2004–2017

**Year**	**Male**					**Female**				
	**Number**	**Crude**	**ASR**	**ASR-L**	**ASR-U**	**Number**	**Crude**	**ASR**	**ASR-L**	**ASR-U**
2004	48	6.22	7.84	5.47	10.21	26	3.32	4.14	2.45	5.83
2005	72	9.14	10.91	8.24	13.58	43	5.39	6.90	4.69	9.11
2006	64	7.96	11.1	8.2	14.00	51	6.27	7.80	5.53	10.07
2007	73	8.90	10.26	7.79	12.73	50	6.04	7.68	5.45	9.91
2008	73	8.72	10.91	8.26	13.56	56	6.64	7.64	5.50	9.78
2009	62	7.27	8.22	6.04	10.40	52	6.06	7.75	5.57	9.93
2010	69	7.94	9.77	7.34	12.20	46	5.26	6.11	4.29	7.93
2011	59	6.66	7.57	5.55	9.59	25	2.81	3.29	1.94	4.64
2012	62	6.92	7.75	5.73	9.77	43	4.79	5.47	3.78	7.16
2013	52	5.73	6.66	4.76	8.56	48	5.30	5.34	3.79	6.89
2014	66	7.19	8.69	6.51	10.87	49	5.36	5.99	4.27	7.71
2015	96	10.35	11.43	9.1	13.76	79	8.57	9.60	7.42	11.78
2016	101	10.76	12.17	9.7	14.64	78	8.38	8.86	6.82	10.90
2017	96	10.12	11.55	9.16	13.94	58	6.18	6.72	4.94	8.50

ASR, Age-standardized incidence rate; ASR-L, Lower limit of 95% CI; ASR-U, Upper limit of 95% CI.

**Figure 2:**
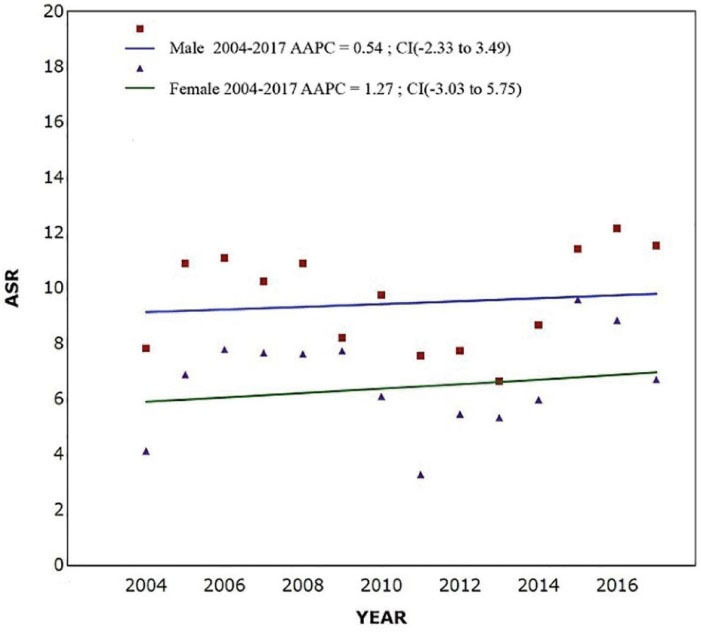
Temporal Variations in Age Standardized Incidence Rate of Leukemia in Golestan, Iran, by Sex, during 2004-2017. AAPC, Average annual percent changes; CI, confidence interval; ACR, age standardized incidence rate.

Figure 2 shows the temporal trends of leukemia over the last decade, suggesting no significant trends in both males (AAPC = 0.54; 95% CI: - 2.33 to 3.49; *P* value = 0.693) and females (AAPC = 1.27; 95% CI: -3.03 to 5.75; *P* value = 0.539), whereas the changes were more considerable in females.

**Figure 3:**
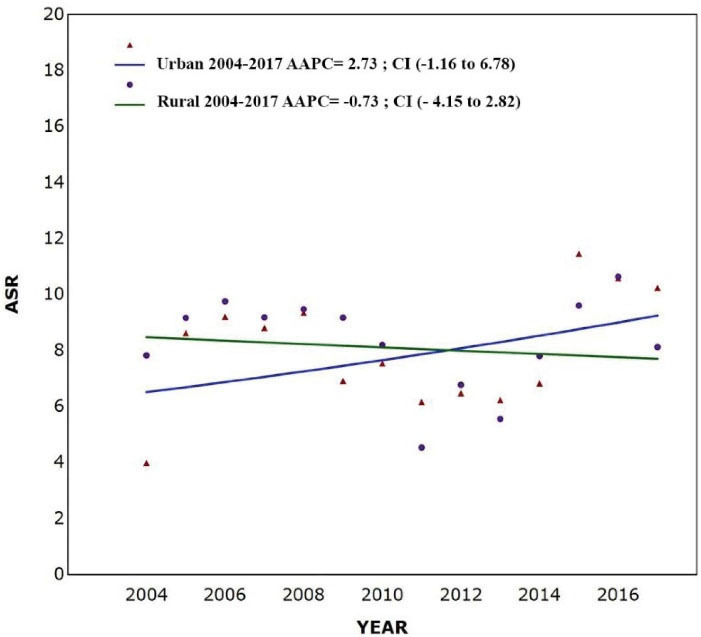
Time trends in Age Standardized Incidence Rate of Leukemia in Golestan, Iran, by Residence Area, during 2004–2017. AAPC, Average annual percent changes; CI, confidence interval; ACR, age standardized incidence rate.

**Figure 4:**
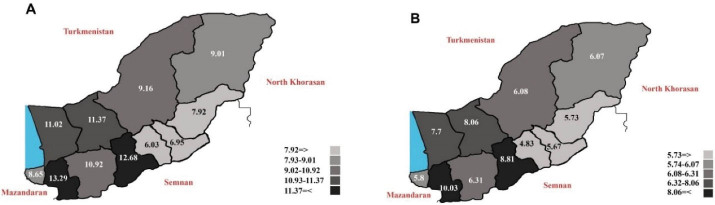
Age Standardized Incidence Rate (Per 100 000 Person-Year) of Leukemia in Golestan Cities (2004–2017). A = Male, B = Female.

 The number (crude rate; ASR) of leukemia cases were 843 (6.78; 8.07) and 845 (7.16; 8.37) per 100 000 person-year in urban and rural regions, respectively. Figure 3 shows the time trends of incidence of leukemia in urban and rural areas of the Golestan province. The findings suggested an increasing trend in the urban population (AAPC = 2.73; 95% CI: -1.16 to 6.78; *P* value = 0.154), while in the rural area, the incidence rates were slightly decreasing (AAPC = -0.73; 95% CI: -4.15 to 2.82; *P* value = 0.658). Figure 4 demonstrates the geographical distribution of leukemia in subdivisions of the Golestan province by gender. The incidence rates of leukemia were highest in central and western parts of the province.

## Discussion

 This study investigated the incidence and time trends of leukemia and MM in the Golestan province of Northern Iran during 2004–2017.

 Our study demonstrated elevated ASRs for leukemia (11.50 and 6.70 per 100 000 in males and females, respectively) in comparison with the national average (8.32 and 5.40 in males and females, respectively).^20^ Based on the GLOBOCAN 2018, the Global incidence rates of leukemia in males and females were 6.10 and 4.30, respectively.^12^

 Our findings also suggested that the incidence rates of MM in Golestan were 2.80 and 2.10 per 100 000 in males and females, respectively. Based on a report of the Iranian national cancer registry, the incidence rates of MM were 2.64 and 1.73 per 100 000 in Iranian males and females, respectively.^20^ Based on GLOBOCAN 2018, the global incidence rates of MM were 2.10 and 1.40 in male and females, respectively.^12^ Another study in Iran estimated the incidence rate of MM at 2.00 and 1.20 in males and females, respectively.^21^ The results of a study from China similarly showed that the incidence rate of MM was 1.84 per 100 000 person-years for males and 1.30 for females.^22^ Therefore, according to our results, it seems that the incidence rates of leukemia and MM in our population are higher than national and even the global averages, suggesting the need to study the risk factors and clarify the reasons for these high rates in our population.

 Our results suggested higher incidence rates of leukemia and MM in males than females. This finding is in agreement with a previous report from other populations in Iran and other countries.^23-25^ Increased rates of leukemia and MM in males might be attributable to a higher prevalence of cigarette smoking,^26^ higher body mass index,^27^ occupational exposure,^28^ environmental carcinogens^29^ and, exposure to specific pesticides.^30^

 In light of the results, we noticed an increasing trend in the incidence rate of leukemia in the urban population, which may be due to lifestyle modification and environmental risk factors in urban areas, such as air pollution (especially long-term exposure to traffic‐related air pollution) during recent years.^31,32^ Another study in an industrial area in northern Italy suggested a possible role for environmental air pollution from industrial sites regarding the risk of leukemia in adult populations.^33^ Ease of access to healthcare services and addressing the diagnostic gap of malignancies in urban areas may also be considered as possible explanation for their increasing trends in our urban population. Further studies are needed to clarify the reasons for the increasing trend in the incidence of leukemia in the urban population of Golestan, Iran.

 Based on our findings, the incidence rate of leukemia was higher in central and western parts of the Golestan province. Other studies from Japan,^34^ Spain^35^ and Mexico^36^ similarly suggest geographical disparities in the incidence of leukemia. These differences may be attributed to differences in the prevalence of genetic or environmental factors, including ionizing radiation, non-ionizing radiation, pesticides, parental exposure, and infections.^36^ The higher rate of urbanization and its related risk factors in the western and central part of the province may also partly explain the higher incidence of leukemia in these parts of the Golestan province. Further investigations should be conducted to clarify the reasons for the higher incidence of leukemia in certain areas of the Golestan province.

 In this study, we obtained data on leukemia and MM from the GPCR dataset, which is a high-quality population-based cancer registry and a voting member of the IACR. Benefitting from the high quality GPCR was the major strength of our study. As in other similar population-based cancer registries, data on risk factors were not available in the GPCR dataset. Therefore, we could not clarify the reasons for time trends and geographical disparities in the incidence of leukemia and MM in the Golestan population. This was a limitation of the present study and should be addressed in future projects.

 In conclusion, we found that the incidence rates of leukemia and MM tend to be elevated in Golestan, Northern Iran, compared with national and global averages. Our results showed a male preponderance in leukemia rates as well as elevated rates in central and western parts of Golestan. We found increasing trends in the incidence of leukemia in the urban population. High prevalence of risk factors including cigarette smoking, higher body mass index in males and exposure to occupational and environmental carcinogens including pesticides may partly explain the high rates and increasing trends in the incidence of leukemia and MM in the Golestan province. Further investigations are warranted to clarify and validate the reasons for the geographical disparities and time trends of these malignancies in our population.

## Supplementary Materials

Supplementary file 1 contains Table S1.
