# Supplementary material for: Incidence, Time Trends and Geographical Distribution of Leukemia and Multiple Myeloma in Golestan Province, Northern Iran, 2004–2017
Source: Arch Iran Med. 2022 Jun 1;25(6):360–5. doi: 10.34172/aim.2022.59 (PMC11904270; doi:10.34172/aim.2022.59)
Supplement: Supplementary file 1 — contains Table S1. [file aim-25-360-s001.pdf]

Supplementary file 1

**Table S1. Number, crude rate and age standardized incidence rate (ASR) (per 100,000 person-year) of type of leukemia and MM in Golestan, Iran during 2004 to 2017**											
**Type of cancer**	**Year**	**Male**					**Female**				
		**Number**	**Crude**	**ASR**	**ASR-L**	**ASR-U**	**Number**	**Crude**	**ASR**	**ASR-L**	**ASR-U**
**Lymphoid-Leukemia**	2004	14	1.81	2.11	0.93	3.29	9	1.15	1.62	0.48	2.76
	2005	28	3.55	4.16	2.53	5.79	15	1.88	2.62	1.21	4.03
	2006	38	4.73	6.46	4.26	8.66	15	1.85	2.2	1	3.4
	2007	35	4.27	4.66	3.05	6.27	17	2.05	2.49	1.24	3.74
	2008	33	3.94	5.17	3.29	7.05	24	2.85	3.34	1.91	4.77
	2009	27	3.16	3.4	2.07	4.73	15	1.75	2.28	1.08	3.48
	2010	38	4.37	5.36	3.58	7.14	21	2.4	2.83	1.58	4.08
	2011	17	1.92	2.49	1.26	3.72	5	0.56	0.7	0.07	1.33
	2012	32	3.57	3.95	2.52	5.38	23	2.56	2.97	1.72	4.22
	2013	20	2.21	2.67	1.45	3.89	7	0.77	0.81	0.18	1.44
	2014	21	2.29	2.66	1.46	3.86	16	1.75	1.91	0.95	2.87
	2015	62	6.68	7.7	5.76	9.64	39	4.23	5.14	3.51	6.77
	2016	30	3.2	4	2.53	5.47	23	2.47	2.86	1.66	4.06
	2017	41	4.32	5.1	3.49	6.71	17	1.81	1.97	1.01	2.93
	2004	9	1.17	1.63	0.47	2.79	6	0.77	0.95	0.17	1.73
	2005	15	1.9	2.09	0.97	3.21	10	1.25	1.62	0.54	2.7
	2006	12	1.49	2.04	0.84	3.24	19	2.34	3.06	1.59	4.53
	2007	19	2.32	2.87	1.5	4.24	18	2.17	2.88	1.49	4.27

**Myeloid-Leukemia**	2008	21	2.51	3.18	1.73	4.63	16	1.9	2.1	1	3.2
	2009	18	2.11	2.4	1.2	3.6	18	2.1	2.63	1.4	3.86
	2010	21	2.42	2.97	1.62	4.32	12	1.37	1.48	0.62	2.34
	2011	19	2.14	2.24	1.2	3.28	8	0.9	1.05	0.27	1.83
	2012	17	1.9	2.38	1.18	3.58	11	1.23	1.38	0.54	2.22
	2013	15	1.65	1.67	0.77	2.57	28	3.09	3.02	1.86	4.18
	2014	23	2.51	3.06	1.77	4.35	19	2.08	2.36	1.26	3.46
	2015	23	2.48	2.34	1.36	3.32	17	1.84	1.92	0.98	2.86
	2016	35	3.73	3.92	2.57	5.27	25	2.69	2.9	1.72	4.08
	2017	27	2.85	3.1	1.88	4.32	17	1.81	2.15	1.11	3.19
**Unspecified-Leukemia**	2004	25	3.24	4.1	2.39	5.81	11	1.41	1.58	0.6	2.56
	2005	29	3.68	4.65	2.87	6.43	18	2.26	2.67	1.36	3.98
	2006	14	1.74	2.6	1.15	4.05	17	2.09	2.53	1.26	3.8
	2007	19	2.32	2.73	1.44	4.02	15	1.81	2.31	1.09	3.53
	2008	19	2.27	2.56	1.36	3.76	16	1.9	2.2	1.06	3.34
	2009	17	1.99	2.42	1.2	3.64	19	2.21	2.84	1.51	4.17
	2010	10	1.15	1.45	0.51	2.39	13	1.49	1.79	0.77	2.81
	2011	23	2.6	2.84	1.61	4.07	12	1.35	1.54	0.64	2.44
	2012	13	1.45	1.41	0.61	2.21	9	1	1.11	0.35	1.87
	2013	17	1.87	2.32	1.16	3.48	13	1.44	1.52	0.68	2.36
	2014	22	2.4	2.96	1.69	4.23	14	1.53	1.72	0.8	2.64
	2015	11	1.19	1.38	0.54	2.22	23	2.49	2.54	1.46	3.62
	2016	36	3.84	4.25	2.8	5.7	30	3.22	3.09	1.93	4.25

	2017	28	2.95	3.35	2.08	4.62	24	2.56	2.6	1.52	3.68
**Multiple Myeloma (MM)**	2004	6	0.78	1.43	0.25	2.61	5	0.64	1.2	0.12	2.28
	2005	7	0.89	1.2	0.3	2.1	10	1.25	2.04	0.75	3.33
	2006	10	1.24	2.01	0.72	3.3	8	0.98	1.59	0.43	2.75
	2007	22	2.68	3.84	2.15	5.53	16	1.93	2.96	1.45	4.47
	2008	19	2.27	3.23	1.72	4.74	12	1.42	2.07	0.87	3.27
	2009	7	0.82	1.23	0.27	2.19	14	1.63	2.27	1.04	3.5
	2010	20	2.3	3.23	1.76	4.7	15	1.72	2.25	1.09	3.41
	2011	18	2.03	3.15	1.64	4.66	12	1.35	1.58	0.68	2.48
	2012	18	2.01	2.54	1.34	3.74	16	1.78	2.23	1.11	3.35
	2013	19	2.1	2.62	1.4	3.84	6	0.66	0.78	0.13	1.43
	2014	25	2.73	3.23	1.94	4.52	20	2.19	2.55	1.39	3.71
	2015	28	3.02	3.89	2.38	5.4	17	1.84	1.89	0.97	2.81
	2016	17	1.81	2.42	1.22	3.62	17	1.83	2.17	1.13	3.21
	2017	22	2.32	2.76	1.56	3.96	16	1.7	2.06	1.04	3.08
